# Crimean-Congo Hemorrhagic Fever Virus Endemicity in United Arab Emirates, 2019

**DOI:** 10.3201/eid2605.191414

**Published:** 2020-05

**Authors:** Jeremy V. Camp, Dafalla O. Kannan, Babiker Mohammed Osman, Moayyed Sher Shah, Brigitte Howarth, Tamer Khafaga, Pia Weidinger, Noushad Karuvantevida, Jolanta Kolodziejek, Hessa Mazrooei, Nadine Wolf, Tom Loney, Norbert Nowotny

**Affiliations:** University of Veterinary Medicine Vienna, Vienna, Austria (J.V. Camp, P. Weidinger, J. Kolodziejek, N. Wolf, N. Nowotny);; Al Ain City Municipality, Al Ain, United Arab Emirates (D.O. Kannan, B.M. Osman);; Dubai Desert Conservation Reserve, Dubai, United Arab Emirates (M. Sher Shah, T. Khafaga);; Zayed University, Dubai (B. Howarth);; Mohammed Bin Rashid University of Medicine and Health Sciences, Dubai (N. Karuvantevida, H. Mazrooei, T. Loney, N. Nowotny)

**Keywords:** *Camelus*, dromedary camels, endemic diseases, *Hyalomma* ticks, Crimean-Congo hemorrhagic fever virus, zoonoses, United Arab Emirates, viruses, vector-borne infections, sequencing

## Abstract

We conducted a cross-sectional survey of Crimean-Congo hemorrhagic fever virus (CCHFV) in dromedary camels and attached ticks at 3 locations in the United Arab Emirates. Results revealed a high prevalence of CCHFV-reactive antibodies in camels and viral RNA in ticks and camel serum, suggesting the virus is endemic in this country.

Crimean-Congo hemorrhagic fever virus (CCHFV; order Bunyavirales, family *Nairoviridae*, genus *Orthonairovirus*) is a geographically widespread species of tickborne virus. Enzootic transmission cycles involve livestock (cattle, sheep, goats) and tick species of the genus *Hyalomma* (Acari: *Ixodidae*) (*1*). Spillover into humans typically occurs through tick bites; however, some severe (and even fatal) CCHFV infections have occurred as a result of exposure to blood or tissue from infected animals. The virus is genetically diverse, and evidence indicates that frequent reassortment of viral gene segments occurs, potentially as a result of animal trade between regions of Africa and Asia (*1*,*2*).

Reports of infections in humans during 2016–2019 (*3*–*5*), the outbreak in the United Arab Emirates (UAE) in 1979 (*6*), and the outbreak in Oman during 1994–1995 (*7*) suggest that CCHFV is present in the Arabian Peninsula. However, little is known about enzootic transmission and the frequency of importation into this region. Therefore, we conducted a cross-sectional survey of ticks and dromedary camels in the UAE to determine exposure status and detect active CCHFV infections.

We collected whole blood samples from camels at 3 sites within the UAE that differed in camel use: a family farm, a desert conservation reserve with multiple tour operators, and a large livestock market (Appendix). We found CCHFV antibodies in the serum samples of 67% (84/125) of camels. CCHFV antibody prevalence was highest in older camels (96% in camels >10 years of age), and no difference in antibody prevalence was detected between sexes (68% [51/75] male, 71% [29/41] female) (Appendix Table 1). The prevalence of reactive antibodies differed between sampling locations, potentially because of differences in animal ages at the respective sites.

We removed 314 adult ticks and 33 tick nymphs (0–5 ticks/camel) from camels and identified the species under a stereomicroscope. Most (99%, 311/314) adults were *Hyalomma dromedarii* ticks, and 3 were *H. scupense* ticks. Two pools of adult *H. dromedarii* ticks (1 containing 3 males and the other containing 1 male) from 2 separate camels (both 6-year-old females, one of which was antibody positive) and serum samples from 2 camels (a 3-year-old female and 2-year-old male, both antibody negative) were positive for CCHFV nucleic acid (Appendix Table 2). These 4 camels were all from the livestock market but originated from different regions of the UAE. The 2 camels with CCHFV RNA–positive serum were only briefly at the livestock market (for 1 and 2 days), and the 2 with CCHFV RNA–positive ticks were housed at the market for 7 and 41 days.

We performed 2 conventional reverse transcription PCRs on the RNA-positive serum samples and on each tick from the 2 RNA-positive pools, 1 amplifying a 492-bp portion of the viral small (S) segment and 1 amplifying a 672-bp portion of the viral medium (M) segment (Appendix). We then subjected these PCR products to Sanger sequencing (GenBank accession nos. MN516481–8; Appendix Table 3). The S segment sequences from 3 ticks (from 2 camels) and 2 serum samples were all identical to each other, except for a single synonymous substitution in the sequence from 1 serum sample; these sequences were genetically similar to sequences of isolates from West and South Africa (group III; Figure, panel A). We obtained the M segment sequences from only 3 ticks from 2 camels. These sequences were 85% identical to available sequences in GenBank, and the isolate with the closest identity (AP92, GenBank accession no. DQ211625) was from Greece (Figure panel B). Thus, the 2019 UAE isolates did not fall within previously defined phylogenetic groups (*2*).

**Figure:**
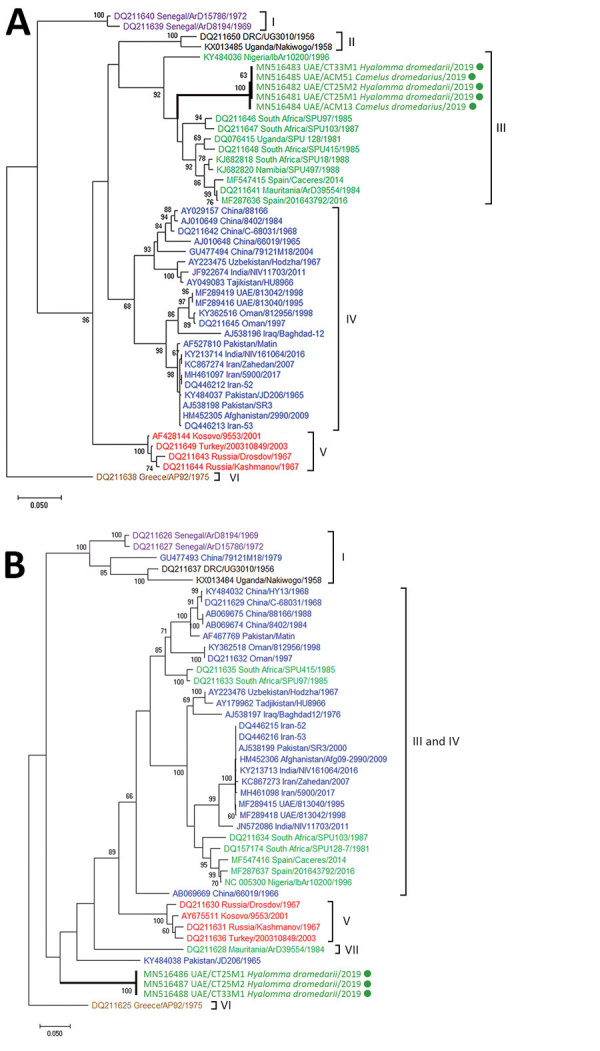
Molecular phylogeny of Crimean-Congo hemorrhagic fever viruses from dromedary camel serum samples and ticks (green circles, thick branches), United Arab Emirates, 2019. A maximum-likelihood analysis of a 492-nt sequence of the viral small (S) segment (A) and 672-nt sequence of the viral medium (M) segment (B) were performed. Viruses are labeled by GenBank accession number, country of origin, isolate name, and year of identification and are colored according to S segment lineages following the group nomenclature (*2*): group I, West Africa 1; group II, Democratic Republic of the Congo; group III, South Africa and West Africa 2; group IV, Asia and the Middle East; group V, Europe and Turkey; group VI, Greece; and group VII (M segment only). Numbers beside branches are bootstrap values from 500 bootstrap replicates; only values >60% are shown. Scale bars indicate number of substitutions per site.

Our data indicate that exposure to CCHFV is common among camels in the UAE, and transmission to camels might be occurring via native infected *H. dromedarii* ticks. A previous survey of UAE livestock that occurred shortly after the 1994–1995 outbreak ruled out camels and camel ticks as CCHFV reservoirs (*7*). Our data might indicate increased transmission activity in the region, potentially explaining the human case in Sharjah, UAE, in August 2019 associated with handling infected meat (*5*). The largest outbreak of CCHFV infection in the UAE (1994–1995) was associated with a high case-fatality ratio (73%) and was limited to abattoir workers (*8*,*9*); however, hospital outbreaks have also previously occurred in the UAE (*6*).

All previously characterized CCHFV isolates from the Arabian Peninsula and the Middle East (including viruses from the UAE and Oman) were genetically similar to each other, clustering together according to the S segment (group IV, Figure panel A). The M segments of the isolates from UAE and Oman were similar to those of viruses from Asia, the Middle East, West Africa, and South Africa (Figure panel B) (*2*,*3*,*7*). Overall, the data suggest that CCHFV is endemic in the UAE, where enzootic transmission cycles involve camels and camel ticks.

Appendix More information on Crimean-Congo hemorrhagic fever virus endemic in United Arab Emirates, 2019.
