# Supplementary material for: Crimean-Congo Hemorrhagic Fever Virus Endemicity in United Arab Emirates, 2019
Source: Emerg Infect Dis. 2020 May;26(5):1019–21. doi: 10.3201/eid2605.191414 (PMC7181925; doi:10.3201/eid2605.191414)
Supplement: Appendix — More information on Crimean-Congo hemorrhagic fever virus endemic in United Arab Emirates, 2019. [file 19-1414-Techapp-s1.pdf]

Article DOI: https://doi.org/10.3201/eid2605.191414

Crimean-Congo Hemorrhagic Fever Virus Endemicity in United Arab Emirates, 2019

Appendix

Supplemental Methods

Three sites were specifically selected within the United Arab Emirates (UAE) that differed in camel use: (i) a family farm with camels primarily raised for racing, breeding, and trading; (ii) tour operators at a desert conservation reserve with camels used for tourism; and (iii) a large high-turnover livestock market with camels used for trading and meat. Whole blood was drawn from camels into serum tubes as part of mandated brucellosis testing. Camel age and sex were recorded. All camels originated within the UAE. Sera were separated from whole blood by centrifugation, and then tested for the presence of Crimean-Congo hemorrhagic fever virus (CCHFV)-reactive antibodies using a commercial ELISA kit (ID Screen® CCHF Double Antigen Multi-species, IDVet).

During blood-taking, ticks were removed from the camels by hand and frozen at -80°C. Adult ticks were later identified to species on dry ice under a stereomicroscope using species descriptions and dichotomous keys (*1–4*). Molecular barcoding was used to confirm selected voucher specimens to species, using PCR to amplify a portion of *cytochrome oxidase I* (*5*). Parasagittal sections were taken from individual ticks with a sterile scalpel blade, the halves were pooled by camel and stadium, and homogenized in DNA/RNA Shield (ZymoResearch) with metal beads using a TissueLyzer (Qiagen). RNA was extracted from tick homogenates and camel serum with a commercial kit (QIAamp viral RNA kit, Qiagen) and tested for the presence of CCHFV nucleic acid using a commercial reverse transcription quantitative PCR (RT-qPCR) assay (RealStar® CCHFV RT-PCR Kit, Altona).

Putative-positive samples were then tested by conventional RT-PCR to amplify a 492 base portion of the viral small (S) segment (using primers F2 and R3 from (*6*)) and a 672 base portion of the viral medium (M) segment (primers CCHFV_3605F, 5’-

CAGAAAGATGTGGCTGCACA; CCHFV_4316R, 5’-TCTCCRTGTGCWGTRACCCT) and subjected to Sanger sequencing. The resulting sequences were aligned to published sequences found in GenBank using Mega7 (version 7.0.26) that were representative of the various genetic lineages of CCHFV (*7*) and for which both S and M segment sequences were available.

References

1. Apanaskevich DA, Schuster AL, Horak IG. The genus *Hyalomma*: VII. Redescription of all parasitic stages of *H.* (*Euhyalomma*) *dromedarii* and *H.* (*E.*) *schulzei* (Acari: *Ixodidae*). J Med Entomol. 2008;45:817–31. PubMed http://dx.doi.org/10.1093/jmedent/45.5.817

2. Apanaskevich DA, Filippova NA, Horak IG. The genus *Hyalomma* Koch, 1844. X. Redescription of all parasitic stages of *H.* (*Euhyalomma*) *scupense* Schulze, 1919 (= *H. detritum* Schulze) (Acari: *Ixodidae*) and notes on its biology. Folia Parasitol (Praha). 2010;57:69–78. PubMed http://dx.doi.org/10.14411/fp.2010.009

3. Asadollah HC, Hosseini R, Tavakoli M, Telmadarraiy Z, Abdigoudarzi M. The Iranian *Hyalomma* (Acari: *Ixodidae*) with a key to the identification of male species. Persian J Acarol. 2013;2:503–29.

4. Estrada-Pena AL. Ticks of domestic animals in the Mediterranean region: a guide to identification of species. Zaragoza, Spain: University of Zaragoza; 2004.

5. Ivanova NV, Zemlak TS, Hanner RH, Hebert PDN. Universal primer cocktails for fish DNA barcoding. Mol Ecol Notes. 2007;7:544–8. http://dx.doi.org/10.1111/j.1471-8286.2007.01748.x

6. Schwarz TF, Nsanze H, Longson M, Nitschko H, Gilch S, Shurie H, et al. Polymerase chain reaction for diagnosis and identification of distinct variants of Crimean-Congo hemorrhagic fever virus in the United Arab Emirates. Am J Trop Med Hyg. 1996;55:190–6. PubMed http://dx.doi.org/10.4269/ajtmh.1996.55.190

7. Deyde VM, Khristova ML, Rollin PE, Ksiazek TG, Nichol ST. Crimean-Congo hemorrhagic fever virus genomics and global diversity. J Virol. 2006;80:8834–42. PubMed http://dx.doi.org/10.1128/JVI.00752-06

Appendix Table 1. Camel sera with antibodies reactive to CCHFV in the United Arab Emirates, 2019 (number positive / number tested, %) measured by commercial ELISA*

	CCHFV-reactive antibodies (no. positive/total tested, %)			
Age class (yr)	Livestock Market	Family Farm	Desert Reserve	TOTAL
< 2	4 / 10	1 / 1	1 / 1	6 / 12, 50%
2-5	11 / 29	1 / 4	2 / 6	14 / 39, 36%
6-10	3 / 4	3 / 3	12 / 15	18 / 22, 82%
> 10	0 / 0	7 / 7	36 / 38	43 / 45, 96%
Unknown	3 / 7	0 / 0	0 / 0	3 / 7, 4%
TOTAL	21 / 50, 42%	12 / 15, 80%	51 / 60, 85%	84 / 125, 67%

*CCHFV, Crimean-Congo hemorrhagic fever virus.

Appendix Table 2. Demographic information and results of serum antibody ELISA (CCHFV-Ab) for samples that tested positive for CCHFV by RT-qPCR*

ID	Sample Type	Sex	Age	Days at Market	CCHFV-Ab
CT25	Tick pool (3 ♂)	F	6	7	Negative
CT33	Tick pool (1 ♂)	F	6	41	Positive
ACM13	Camel serum	F	3	1	Negative
ACM51	Camel serum	M	2	2	Negative

*Information for tick pools refers to host status. Ab, antibody; CCHFV, Crimean-Congo hemorrhagic fever virus; RT-qPCR, reverse transcription quantitative PCR.

Appendix Table 3. Results of molecular diagnostics for positive camel serum and camel tick samples*

ID	Altona RT-qPCR	RT-PCR S segment	RT-PCR M segment
CT25	22.5	MN516481, MN516482	MN516486, MN516487
CT33	23.9	MN516483	MN516488
ACM13	>40	MN516484	Negative
ACM51	36.7	MN516485	Negative

*For screening, RT-qPCR kit was used and Ct values are given. The products from follow-up conventional RT-PCR of putative positive samples were sequenced and deposited in GenBank (accession numbers given). Ct, cycle threshold; ID, identification; M, medium; RT-PCR, reverse transcription PCR; RT-qPCR, reverse transcription quantitative PCR; S, small.
